# Spontaneous spinal cord infarction: a systematic review

**DOI:** 10.1136/bmjno-2024-000754

**Published:** 2024-05-27

**Authors:** Maria Gharios, Vasilios Stenimahitis, Victor Gabriel El-Hajj, Omar Ali Mahdi, Alexander Fletcher-Sandersjöö, Pascal Jabbour, Magnus Andersson, Claes Hultling, Adrian Elmi-Terander, Erik Edström

**Affiliations:** 1 Department of Clinical Neuroscience, Karolinska Institutet, Stockholm, Sweden; 2 Department of Rehabilitation, Furuhöjden Rehab Hospital, Täby, Sweden; 3 Department of Neurological Surgery, Thomas Jefferson University Hospital, Philadelphia, Pennsylvania, USA; 4 Department of Neurobiology, Care Sciences and Society, Karolinska Institutet, Stockholm, Sweden; 5 Capio Spine Center Stockholm, Löwenströmska Hospital, Upplands-Väsby, Sweden; 6 Department of Medical Sciences, Örebro University, Örebro, Sweden; 7 Department of Surgical Sciences, Uppsala University, Uppsala, Sweden

**Keywords:** STROKE

## Abstract

**Background and objectives:**

Spontaneous spinal cord infarction (SCInf) is a rare condition resulting in acute neurological impairment. Consensus on diagnostic criteria is lacking, which may present a challenge for the physician. This review aims to analyse the current literature on spontaneous SCInf, focusing on epidemiology, the diagnostic process, treatment strategies and neurological outcomes.

**Methods:**

The study was performed in accordance with a previously published protocol. PubMed, Web of Science and Embase were searched using the keywords ‘spontaneous’, ‘spinal cord’, ‘infarction’ and ‘ischaemic’. The eligibility of studies was evaluated in two steps by multiple reviewers. Data from eligible studies were extracted and systematically analysed.

**Results:**

440 patients from 33 studies were included in this systematic review. Analysis of vascular risk factors showed that hypertension was present in 40%, followed by smoking in 30%, dyslipidaemia in 29% and diabetes in 16%. The severity of symptoms at admission according to the American Spinal Injury Association (ASIA) Impairment Scale was score A 19%, score B14%, score C36% and score D32%. The mean follow-up period was 34.8 (±12.2) months. ASIA score at follow-up showed score A 11%, score B 3%, score C 16%, score D 67% and score E 2%. The overall mortality during the follow-up period was 5%. When used, MRI with diffusion-weighted imaging (DWI) supported the diagnosis in 81% of cases. At follow-up, 71% of the patients were able to walk with or without walking aids.

**Conclusion:**

The findings suggest a significant role for vascular risk factors in the pathophysiology of spontaneous SCInf. In the diagnostic workup, the use of DWI along with an MRI may help in confirming the diagnosis. The findings at follow-up suggest that neurological recovery is to be expected, with the majority of patients regaining ambulation. This systematic review highlights gaps in the literature and underscores the necessity for further research to establish diagnostic criteria and treatment guidelines.

**WHAT IS ALREADY KNOWN ON THIS TOPIC**

- Spinal cord infarction (SCInf) is a rare condition leading to significant neurological impairment. However, definitive diagnostic criteria and treatment guidelines are still lacking.

**WHAT THIS STUDY ADDS**

- This systematic review provides a comprehensive summary of spontaneous SCInf, focusing on its epidemiology, clinical presentation, risk factors, diagnosis, treatment and outcomes.

**HOW THIS STUDY MIGHT AFFECT RESEARCH, PRACTICE OR POLICY**

- This study summarises the available literature regarding the diagnosis, treatment and prognostic factors of spontaneous SCI. It also offers a flowchart with suggested diagnostic and treatment strategies, as well as expert recommendations. Furthermore, it underscores the need for further research in order to establish definitive diagnostic criteria and treatment strategies.

## Introduction

 Spinal cord infarction (SCInf) constitutes approximately 6% of all acute myelopathic syndromes^1^ and 1.2% of all strokes.^2^,^5^ It arises either within a periprocedural context, attributed to aortic disease and repair surgery,^6 7^ or as a spontaneous pathology.^8^,^10^ Like cerebral strokes, the occurrence of spontaneous SCInf has been ascribed to the interplay between various vascular risk factors such as diabetes, hypertension and hyperlipidaemia.^11 12^ Yet, the exact aetiology behind spontaneous SCInf has not been clarified.^11^

The clinical presentation of SCInf ranges from transient sensory disturbances to severe paraplegia or tetraplegia.^1 13^ Nonetheless, acute severe back pain in approximately 70% of the cases,^14^ followed by a prompt debut of neurological deficits, are described as distinguishing features.^15^ Additionally, impairment of autonomic functions along with bladder and bowel dysfunction may occur.^6 16 17^

The differential diagnosis presents a serious challenge since the acute symptomatology in SCInf is analogous to many other neurological conditions, such as inflammatory myelopathies, multiple sclerosis, malignancy and infectious myelopathies.^6 16^ MRI plays an important role in the diagnostic process. Recently, Zalewski *et al*^18^ have proposed criteria for the diagnosis of both spontaneous and periprocedural SCInf based on clinical, radiological and cerebrospinal fluid (CSF) findings.

Established treatment protocols are lacking. Management strategies reflect those used in cerebral stroke^19^ with antiplatelet therapy, management of cardiovascular risk factors and intensive neurological rehabilitation.

While a rare diagnosis, SCInf has devastating consequences for the individual, and the limited knowledge on the aetiology, diagnostics and treatment options prompts further research. In that context, this systematic review aimed to highlight the current knowledge on spontaneous SCInf and provide an overview of the existing data.

## Materials and methods

This systematic review is in accordance with the Preferred Reporting Items for Systematic Reviews and Meta-Analyses^20^ guidelines (online supplemental file 1). The review protocol was registered within the International Prospective Register of Systematic Reviews (registration ID: CED42023393241; registration date: 24/02/2023). The study protocol was published.^21^

### Databases and search strategy

Electronic search engines, including PubMed, Web of Science and Embase, were searched using different combinations of the following keywords: ‘spontaneous’, ‘spinal cord’, ‘infarction’ and ‘ischaemic’. The detailed search strategy for each of the search engines is included in online supplemental file 2.

### Inclusion criteria

#### Types of studies

All peer-reviewed and original studies, written in English and available in the PubMed, Embase or Web of Science databases from inception and onwards, will be eligible for inclusion.

#### Types of participants

All patients with spontaneous spinal cord infarctions will be included, regardless of age, ethnicity and sex.

#### Types of outcome measurements

Epidemiological data such as age, sex and socioeconomic factors, risk factors, diagnosis and management strategies, outcomes and predictors will all be addressed. Furthermore, outcome parameters, including pathological mechanisms, quality of life and mortality, will be explored with sufficient data.

### Exclusion criteria

Non-original publications such as reviews, editorials and letters to the editor will be disregarded, along with conference abstracts and case reports. Non-spontaneous cases of SCInf occurring after clear inciting events, such as surgery, trauma or hypovolemic shock, will be disregarded and excluded from the analysis. Studies containing both spontaneous and non-spontaneous SCInf cases will only be retained if data on spontaneous cases can be separately extracted. Studies only addressing SCInfs secondary to vertebral artery dissections will also be excluded, as this topic has specifically been addressed in a previous systematic review.^22^

### Study selection

Searches across all search engines from inception until 2023 yielded a total of 743 publications. After duplicate removal, the remaining studies were transferred to Rayyan, where the selection process took place.^23^ The studies were first screened based on titles and abstracts by two independent and blinded reviewers (VS and MG). Then, full-text articles were assessed by the same independent and blinded reviewers. Inter-reviewer conflicts were resolved through discussion and a third reviewer (AET) was consulted as needed.

### Data extraction and synthesis

Data from selected records was extracted using a predefined extraction template, preliminary including (1) general information—title, first author, journal, publication year, etc; (2) study characteristics—study type, sample size, follow-up time, etc; (3) patient characteristics and epidemiology—age, sex, spinal segment involved, presenting symptoms and neurological function, etc; (4) diagnosis and treatment characteristics—diagnostic modalities, treatment strategy, etc; and (5) outcomes—neurological outcomes, predictors of outcome, quality of life, etc. The collaboration of multiple reviewers will be sought to achieve a thorough extraction of the data. The final work will be assessed and cross-checked to prevent any errors.

### Risk of bias and evidence certainty assessment

The risk of bias was assessed using the Newcastle-Ottawa scale (NOS), a scoring system designed for observational studies that allows a maximum of nine points per study. The results of this assessment are provided (online supplemental file 3).

## Results

**Figure 1:**
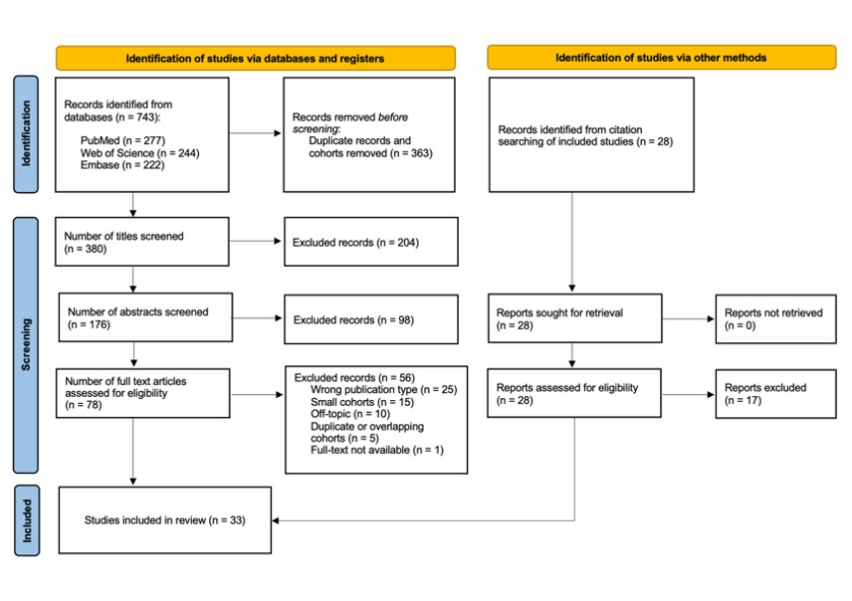
Preferred Reporting Items for Systematic Reviews and Meta-Analyses 2020 flow diagram.

The search strategy yielded 743 studies across three different search engines. Screening of these studies as well as an additional 28 identified from reference list searching resulted in the final inclusion of 33 studies involving 440 patients with spontaneous SCInf (figure 1). For studies with overlapping cohorts, the data were only considered once to avoid duplicate data. Baseline characteristics are presented in table 1.

**Table 1:** Baseline characteristics

Study title	Study ID	n	Spontaneous SCInf (%)	Mean age	% Males
MR imaging of spontaneous spinal cord infarction.	Elksnis (1991)^13^	3	3	50	0%
Spontaneous thoracolumbar spinal cord infarction: report of six cases.	Monteiro (1992)^30^	6	6	57	50%
MR imaging of spinal cord and vertebral body infarction.	Yuh (1992)^66^	12	3	53	100%
Spinal infarction. A follow-up study.	Pelser (1993)^67^	10	8	58	62.50%
Spinal cord infarction: etiology and outcome.	Cheshire (1996)^34^	44	4	63	Not specified
Vertebral body infarction as a confirmatory sign of spinal cord ischemic stroke: report of three cases and review of the literature	Faig (1998)^1^	3	3	53	33.30%
Non-traumatic ischaemic myelopathy: a review of 25 cases.	Kim (1988)^40^	25	7	68	100%
Spinal cord infarction: MR imaging and clinical features in 16 cases.	Weidauer (2002)^39^	16	8	66	62.50%
Diffusion-weighted MR imaging (DWI) in spinal cord ischemia.	Thurnher (2006)^50^	6	3	Not specified	33.30%
Vertebral body signal changes in spinal cord infarction: histopathological confirmation.	Srikanth (2007)^26^	5	5	50	80%
Spinal cord infarction in Chinese patients. Clinical features, risk factors, imaging and prognosis.	Cheng (2008)^31^	22	15	58	40%
Clinical evaluation of patients with spinal cord infarction in Mashhad, Iran.	Ghandehari (2010)^25^	14	7	41	14.30%
Clinical core symptoms of posterior spinal artery ischemia.	Struhal (2011)^6^	4	3	68	66.70%
Acute spinal-cord ischemia: evolution of MRI findings.	Alblas (2012)^33^	5	3	60	33.30%
Retrospective case series of outcomes following spinal cord infarction.	New (2012)^10^	44	11	Not specified	36.40%
Recovery after spinal cord infarcts: long-term outcome in 115 patients.	Robertson 2012^36^	115	24	64	Not specified
Survival following spinal cord infarction.	New (2013)^8^	44	11	Not specified	36.40%
Three unique presentations of atraumatic spinal cord infarction in the pediatric emergency department.	Spencer (2014)^9^	3	3	11	33.30%
Nontraumatic spinal cord ischaemic syndrome.	Rigney 2015^7^	8	4	58	25%
Diagnostic and prognostic relevance of magnetic resonance imaging and electrophysiological findings in acute spinal ischemia	Artemis (2017)^16^	10	10	63	50%
Childhood idiopathic spinal cord infarction: description of 7 cases and review of the literature.	Bar (2017)^17^	7	7	14	14.30%
Delayed hospital presentation and neuroimaging in non-surgical spinal cord infarction.	Pikija (2017)^48^	39	27	68	51.90%
A population-based study of the incidence of acute spinal cord infarction.	Qureshi (2017)^35^	8	5	64	40%
Spinal cord infarction: clinical and radiological features.	Yadav 2018^24^	17	12	28	58.30%
Spontaneous posterior spinal artery infarction: an under-recognized cause of acute myelopathy.	Zalewski (2018)^68^	15	15	Not specified	40%
Characteristics of spontaneous spinal cord infarction and proposed diagnostic criteria.	Zalewski (2019)^18^	133	133	60	46.62%
Acute spontaneous spinal cord infarction: utilisation of hyperbaric oxygen treatment, cerebrospinal fluid drainage and pentoxifylline.	Ashton (2020)^28^	13	13	55	53.80%
Spinal cord transient ischemic attack: Insights from a series of spontaneous spinal cord infarction.	English (2020)^15^	133	133	60	46.60%
Etiology and outcomes of spinal cord infarct: a case series from a level 1 trauma center.	Ge (2020)^5^	30	6	Not specified	Not specified
Spinal cord infarction: a single center experience and the usefulness of evoked potential as an early diagnostic tool.	Park (2020)^37^	14	13	65	61.50%
Serum neurofilament to magnetic resonance imaging lesion area ratio differentiates spinal cord infarction from acute myelitis.	Sechi (2021)^29^	48	20	56	50%
Spontaneous spinal cord infarction in Austria: a two-center comparative study.	Pikija (2022)^14^	88	71	64	45.10%
Long-term outcomes following periprocedural and spontaneous spinal cord infarctions: a population-based cohort study	Stenimahitis (2023)^38^	57	30	65	53.30%

Sex was specified for 406 patients, of whom 48% were male. A pooled mean age of 58.7±3.96 was calculated from 26 studies on 420 patients,^16 7 9 13 16 17 24^,^39^ while two studies^14 18^ only provided the median ages of 60 and 64 years, respectively. Information on vascular risk factors was present in 17 studies on a total of 264 patients.^16 7 9 13 16 18 24 28 30 32 33 35 37^,^40^ Hypertension, identified in 40% of patients, was notably the most common risk factor, followed by smoking (30%), dyslipidaemia (29%) and diabetes (16%), while 28% had no reported vascular risk factors (table 2).

**Table 2:** Patient demographics and vascular risk factors

Total number of patients included	440
Demographics	
Male sex	195 (48%)
Age	n=420
Mean±SD	58.7±3.96
Vascular risk factors	n=264
Hypertension	106 (40%)
Dyslipidaemia	77 (29%)
Diabetes	41 (16%)
Smoking	79 (30%)
Atrial fibrillation	14 (5.3%)
History of stroke or transient ischaemic attack	9 (3.4%)
History of ischaemic heart disease	25 (9.5%)
Peripheral vascular disease	12 (4.5%)
Obesity	3 (1.1%)
No vascular risk factors	75 (28%)

Nineteen studies presented information on the clinical presentation of patients with spontaneous SCinf (table 3).^16^,^9 13 14 16^ The presenting symptoms reported most frequently were motor deficits (92%), sensory deficits (85%), autonomic dysfunction (76%) and pain (70%). Neurological function on admission was reported in eight studies on 106 patients.^5 7 8 14 16 28 35 38^ However, for 11 patients, the individual American Spinal Injury Association (ASIA) Impairment Scale score was not provided, as the authors only mentioned that nine patients had an ASIA score of A, B or C and two had an ASIA score of D.^8^ Consequently, individual ASIA scores were reported for 95 patients. Among them, 18 patients (19%) had an ASIA score of A, 13 (14%) had an ASIA score of B, 34 (36%) had an ASIA score of C and 32 (32%) had an ASIA score of D. None of the patients were neurologically intact on admission. Furthermore, information on the time to nadir in terms of neurological function was found in four studies on 182 patients.^17 18 24 38^ Of these, 148 (81%) reached nadir within 12 hours, 20 (11%) between 12 and 24 hours and 14 (7.7%) after 24 hours.

**Table 3:** Symptoms and neurological function on admission

Symptoms	N (%)
Motor deficits (n=336)	310 (92%)
Sensory deficits (n=307)	260 (85%)
Pain (n=318)	223 (70%)
Bladder and bowel dysfunction (n=315)	239 (76%)
American Spinal Injury Association Impairment Scale Score (n=95)	
A	18 (19%)
B	13 (14%)
C	34 (36%)
D	30 (32%)
Neurologically intact	0 (0%)
Time to nadir	(n=182)
<12 hours	148 (81%)
12–24 hours	20 (11%)
>24 hours	14 (7.7%)

MRI was used in the assessment of 371 patients. Two hundred and seventy-nine patients showed pathological MRI findings at the initial MRI. Information regarding the time to the initial MRI was provided for 162 patients. Most of these patients (90%) were examined after 1 day, mainly between 1 and 2 days. A minority (10%) was examined within 1 day of presentation. In 92 patients, the initial MRI was normal. For 52 of these patients, imaging was performed within the first 24 hours after symptoms onset, for two after 1 day, and for the remainder, this information was not provided. In 90 patients with an initially normal MRI, a repeat MRI performed 1.5–42 days after admission showed abnormalities consistent with SCInf in 83 patients. Only 87 patients had diffusion-weighted imaging (DWI) performed, revealing diffusion restriction in 71 of them (82%). Regarding the affected spinal levels, the most involved levels were thoracic (33%), followed by cervical (24%), thoracic through lumbar (26%), cervical through thoracic (13%), isolated conus (5%) and cervical through conus (0.5%) (table 4).

**Table 4:** MRI findings

Patients with positive initial MRI	n=279
Time to MRI	
Between 0 and 1 day	26
More than 1 day	146
Not stated	107
Patients with negative initial MRI	n=92
Time to initial MRI	
Between 0 and 1 day	52
More than 1 day	2
Not stated	38
Time to second MRI	n=90
Between 1 and 4 days	22
More than 4 days	30
Not stated	38
Inconclusive MRI findings	7
MRI findings suggestive of SCinf	83
Lesion levels	n=345
Cervical	81 (24%)
Thoracic	113 (33%)
Cervical through thoracic	43 (13%)
Thoracic through lumbar	88 (26%)
Isolated conus	18 (5.2%)
Cervical through conus	2 (0.5%)

ScInf, spinal cord infarction.

Results of the CSF analysis were reported in 13 studies on 174 patients.^17 9 13 16^,^18 30 33 34 36^ The analysis was normal in 70 (40%) of these patients, while high protein levels were the most common pathological finding seen in 85 (49%) patients. Oligoclonal bands were reported in two patients (1%) (table 5).

**Table 5:** Results of the CSF analysis in patients with spontaneous SCInf

CSF analysis	Number of patients=174
Normal	70 (40%)
High protein	85 (49%)
Pleocytosis	21 (12%)
Supernumerary oligoclonal bands	2 (1.1%)

CSF, cerebrospinal fluid; ScInf, spinal cord infarction.

Data on the status of patients at follow-up were obtained from 20 studies (table 6).^16^,^9 13 16^ The pooled mean follow-up time, calculated from 16 studies on 143 patients, was 35 months.^16 7 9 13 17 28 30^,^32 34^ The median follow-up time was presented in two studies and ranged from 1 to 1.9 months.^16 18^ In one study on six patients, the follow-up period ranged from 15 to 41 weeks.^24^ One study did not mention follow-up duration.^33^ Among the patients with information regarding ambulation (n=204), the majority, 42%, were independently ambulatory, 29% were ambulatory with aids, 29% were wheelchair dependent and only a single patient (0.5%) was bedridden. ASIA scores were recorded for 190 patients. Twenty-one (11%) patients had an ASIA score of A, six (3%) B, 31 (16%) C, 129 (67%) D and 4 (2%) E. In seven studies on 29 patients, 23 were reported to have motor deficits at follow-up without specifying the ASIA. Twelve patients had sensory deficits (n=17), and 33 patients had autonomic dysfunction (n=55). The mortality rate among patients with follow-up was calculated to be 5% (16/297).

**Table 6:** Follow-up

Total number of patients with available follow-up	297
Mean follow-up period (months)±SD	34.8±12.2
American Spinal Injury Association Impairment Scale at follow-up	n=190
A	21 (11%)
B	6 (3.2%)
C	31 (16%)
D	128 (67%)
E	4 (2.1%)
Motor deficits (n=215)	209 (97%)
Motor deficits, no American Spinal Injury Association Impairment Scalereported (n=29)	23 (79%)
Sensory deficits (n=17)	12 (71%)
Autonomic dysfunction (n=55)	33 (60%)
Ambulation at follow-up (n=204)	
Wheel-chair dependent	59 (29%)
Ambulatory with aids	59 (29%)
Independently ambulatory	85 (42%)
Bed ridden	1 (0.5%)

Before the diagnosis of SCInf was established, the most common treatment was corticosteroids (n=77). Other, less frequently used treatments were intravenous immunoglobulin (n=18), plasma exchange (n=12), thrombolysis (n=2), azathioprine (n=1), mycophenolate (n=1) and rituximab (n=1). After establishing a diagnosis of spinal cord infarction, 193 patients received antiplatelet therapy, 12 anticoagulation and two thrombolysis.

In two studies, young age at onset was a predictor of adverse outcomes.^14 31^ In the first study, younger age at onset (<55 years) was statistically correlated with poor motor recovery.^31^ In the second study, bed-ridden patients on discharge were more likely to be younger (median 57 years, IQR=53–61), compared with other functional conditions (wheelchair, able to walk with help, self-ambulatory) (median 63 years, IQR=55–73).^14^ In two other studies, age could not be associated with mortality.^8 38^

Three studies compared the outcomes of patients with SCInf of different aetiologies and found no statistical difference in the 1-year and 5-year survivals.^8 17 38^ Patients with spontaneous SCInf had better outcomes with improved ASIA scores and were more likely to be ambulatory at follow-up compared with patients with periprocedural SCInf.^38^ In a paediatric cohort, motor recovery was better in idiopathic SCInf.^17^ The latter study also noted that, compared with other aetiologies, spontaneous SCInf was more commonly the result of an insult to the anterior territory of the spinal cord.^17^

In a study analysing MRI localisation and SCInf outcomes, cervical lesions with anterior cord syndrome were associated with a better outcome than those with multiple-level lesions.^14^ In another study, there was no association between lesion level and survival.^8^ Other outcome predictors were hyperlipidemia and severe initial weakness, judged by low scores (≤2) on the Medical Research Council scale, both of which correlated with a poor outcome. Other vascular risk factors (such as diabetes, hypertension, heart/aortic disease and previous cerebral stroke) and bladder dysfunction did not reach statistical significance.^31^

Two studies compared clinical, imaging and electrophysiological findings between patients with SCInf and those with acute transverse myelitis.^29 37^ Compared with patients with transverse myelitis, patients with SCInf had significantly higher neurofilament light protein serum levels,^29^ more prolonged tibial somatosensory evoked potential latency and shorter lesion length on MRI.^41^ These studies suggested the use of new tools to aid in the diagnosis of SCInf. Sechi *et al* demonstrated that SCInf can be accurately distinguished from acute myelitis by the ratio between NFL and the largest sagittal lesion area on MRI.^29^ Park *et al* suggested the use of evoked potentials as a confirmatory test for an appropriate diagnosis of SCInf.^37^ Another study investigating the utility of electrophysiological studies in outcome prediction showed an association between ASIA score of E at follow-up and normal motor evoked potentials (MEPs). There was a trend for an association between unfavourable outcomes (ASIA score ≤C) and pathological MEP findings, but it did not reach significance.^16^

## Discussion

While SCInf constitutes only a small part of myelopathic syndromes and an even smaller part of all stroke syndromes, its effects may be devastating. To improve outcomes, efforts towards improved diagnostic strategies are needed. In 2019, Zalewski *et al* proposed diagnostic guidelines for SCInf.^18^ Diagnostic categories are used to indicate the quality of the supporting findings. Thus, a definitive diagnosis of SCInf rests on typical clinical and MRI findings.

Currently, treatments focus on managing cardiovascular risk factors recognised in stroke. Although SCInfs share many similarities with cerebral strokes, they remain distinct entities.

Two aetiologies of SCInf have been recognised: periprocedural and spontaneous SCInfs. Periprocedural SCInf often occurs as a complication of vascular surgery, affecting the blood supply to the spinal cord. The remainder of SCInf is spontaneous, with pathophysiology resembling that of cerebral strokes. Since knowledge on the diagnosis, treatment and prevention of spontaneous SCInf is limited, this review aims to provide a comprehensive overview of the current knowledge on spontaneous SCInf.

### Risk factors

In our pooled cohort of patients with spontaneous SCInf, at least one vascular risk factor was reported in 72% of patients, with the two most common being hypertension and smoking. Proper management of well-recognised cardiovascular risk factors, such as dyslipidaemia, diabetes, hypertension and smoking, is essential for the primary and secondary prevention of stroke, and arguably so, for SCInfs.^42^,^44^ However, the impact of cardiovascular risk factors on the pathophysiology of spontaneous SCInf is yet to be fully understood.^45^

### Diagnostics

MRI remains the most important tool in establishing a diagnosis of SCInf. Distinct diagnostic findings on MRI include bilateral hyperintense lesions in the anterior horns (owl’s eyes) on transverse sections, pencil-like hyperintensities on sagittal sequences, and hyperintensities corresponding to the anterior spinal artery. In this pooled analysis, MRI was performed in 371 patients, with positive findings in 279. Most patients were examined within 48 hours and only a small part of them (10%) were examined within the first 24 hours. Reportedly, the sensitivity of early scans is extremely low.^46 47^ Up to half of T2-weighted imaging may not depict any spinal cord lesions within the first 24 hours after symptom onset.^14 48^ In our pooled analysis, we found that one-fourth of the initial scans could not support a definite diagnosis. In patients with an initially normal MRI, 92% had findings consistent with SCInf on a repeat MRI performed 1.5–42 days later. Overall, the relatively high positive predictive value of MRI in the total pooled cohort strengthens the importance of MRI in the diagnostic workup of SCInf. While the diagnostic value of early conventional MRI scans may be questioned in this context, it is important to consider the role of these scans in ruling out other differential diagnoses that may warrant other treatments in the acute phase, while keeping in mind that delayed scans may provide more conclusive results in the diagnosis of SCInf.

On another note, DWI is known to be superior as compared with conventional MRI in detecting early ischaemic lesions in stroke patients.^49^ Similarly, DWI may improve the diagnostic accuracy of MRI in SCInf.^50^ However, several anatomical and physiological aspects of spinal imaging complicate the use of DWI in this instance. These include the heterogeneity of the spinal column structures, the variability of blood supply and the pulsatile movement of the CSF and spinal cord.^24 51^ Nonetheless, there is limited data addressing the utility of DWI in the diagnostic workup of SCInf, with only 87 patients receiving DWI identified in this review. Regardless, the use of DWI in the workup of SCInf may assist in establishing a definite diagnosis and facilitate the elimination of other differentials.

### Treatment

This review revealed that 41% of the patients were treated with corticosteroids based on the clinical suspicion of myelitis and prior to the establishment of a definite spontaneous SCInf diagnosis. The use of corticosteroids for the treatment of SCInfs, in an attempt to lower oxidative stress, lacks supporting evidence and has mainly been advocated by a few case reports.^52^,^54^ Corticosteroids may carry severe side effects, which ought to limit their use prior to the definitive establishment of a diagnosis. Similarly, the use of intravenous thrombolysis was also limited to a small number of patients, precluding definitive conclusions regarding the usefulness of this treatment option.^55^

Data regarding the treatment approach after the establishment of a definite SCInf diagnosis revealed that antiplatelet therapy was initiated in most of the cases (93%). Anticoagulation was prescribed in a limited number of cases (6%) and in two cases (1%) thrombolysis was administered. Nonetheless, the efficacy of these treatment modalities in preventing further deterioration is unknown due to the lack of controlled studies. Strategies such as mean arterial pressure elevation and lumbar CSF drainage,^56^ aiming to enhance spinal cord perfusion, have been used in the context of periprocedural SCInf. However, the role of these approaches in the management of spontaneous SCInf is poorly investigated.

### Neurological recovery and ambulation

The recovery from a spinal cord injury is a challenging process and appropriate patient support and guidance are of utmost importance. Both physical and psychological aspects must be addressed. The complexity of spinal cord rehabilitation and a great need for individualised care suggest the need for specialised healthcare providers. Available studies emphasise the importance of managing secondary complications and an approach aiming to facilitate the individual’s reintegration into the community.^57 58^ Ideally, rehabilitation should begin as soon as acute management allows, but delayed rehabilitation also promotes significant improvements and neurological recovery.^59^

Neurological function recovers to some degree during the recovery period after a SCInf,^13 60^ and the potential for recovery seems to be greater for spontaneous compared with periprocedural SCInf.^30 38 61 62^ Based on the pooled analysis, the proportion of patients with severe spinal cord symptoms, ASIA scores A or B, was reduced by 18% at follow-up. The proportion of patients with mild deficits, ASIA scores C or D, increased by 38% at a mean follow-up of 35 months, and there were four patients with complete neurological recovery (ASIA score E).

The pooled data analysis showed that 71% of patients were able to walk with or without walking aids after an average follow-up of 35 months. Recovery of ambulation is reported at different frequencies in different studies and seems to reflect the relative contributions of spontaneous and periprocedural cases.^5 34 63^ Similarly, spontaneous SCInf were associated with a greater potential for ambulatory recovery as opposed to periprocedural ones.^5 63^

### Mortality

During the calculated mean follow-up of two and a half years, mortality was estimated at 5%. This is considerably lower than previous estimates of 22%–23% in mixed cohorts of spontaneous and periprocedural cases.^36 61 64^ In support of the findings from our pooled analysis, Nedeltchev *et al* reported a mortality rate of 9% in a mixed cohort where only 16% were periprocedural cases.^63^ The mortality data thus supports the finding that spontaneous SCInf is associated with lower mortality and improved outcomes compared with periprocedural cases, and long-term strategies are of great importance.^38^

### Limitations

The limitations of this review mainly derive from the inherent limitations of the articles included, including small sample sizes, intermediate to high risks of bias and observational designs on retrospective cohorts or case series. The heterogeneity of the data did not permit a quantitative meta-analysis and the generalisability of the results is hence limited. In addition, the studies included also reflect the current lack of definitive diagnostic criteria.

## Conclusions and future perspectives

**Figure 2:**
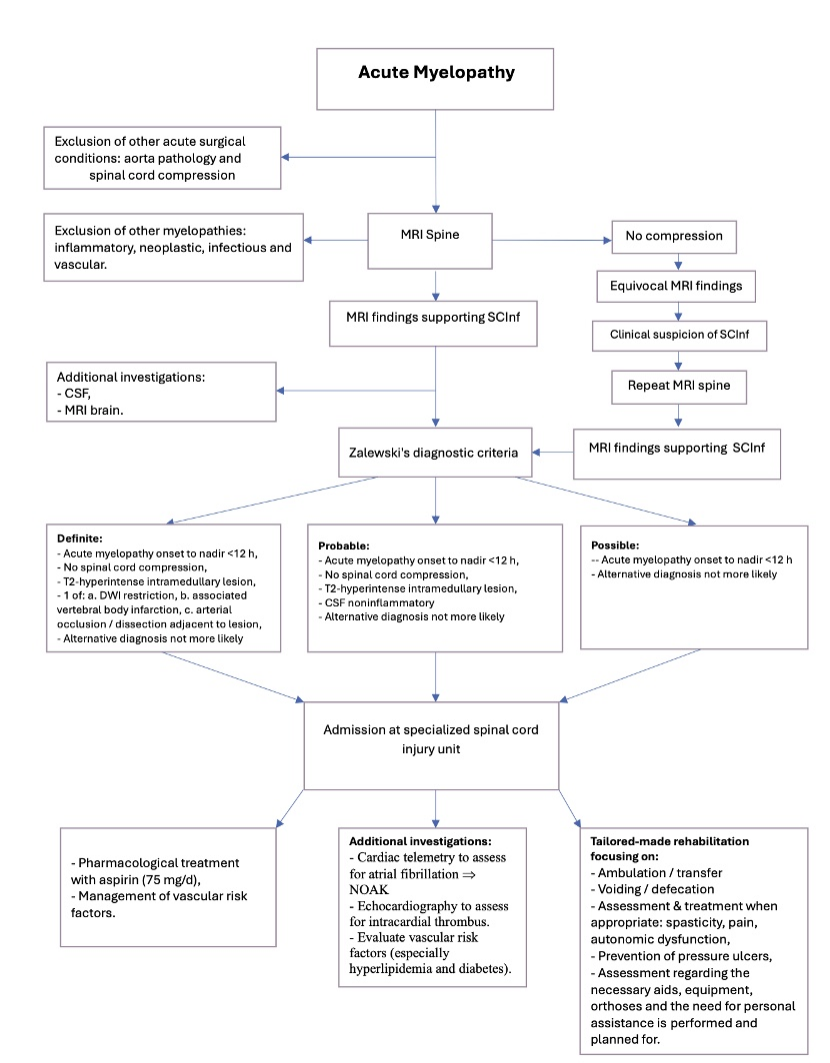
Proposed flow chart for the management of patients with acute myelopathy. ScInf, spinal cord infarction.

Spontaneous SCInf is a rare and often misdiagnosed condition. The multitude of diagnostic alternatives and the lack of definitive diagnostic guidelines and treatment protocols provide an impetus for continued research. Diagnostic criteria for SCInf, such as those proposed by Zalewski *et al*,^18^ should be integrated into the structured management of patients with acute myelopathy (figure 2). A systematic and uniform definition and management of SCInf would provide a foundation for continued clinical and scientific efforts. Currently, treatment is limited to secondary preventive measures. A better understanding of the pathological pathways preceding a spontaneous SCInf could perhaps allow the identification and treatment of individuals at risk. Continued research into the role of cardiovascular risk factors is essential.

Pharmacological treatment with platelet aggregation inhibitors in the aftermath of spontaneous SCInf is recommended, but the efficacy remains unclear. Only four instances of thrombolysis were identified. The difficulties in rapidly establishing a definitive diagnosis may partly explain why thrombolysis is so rarely used. However, the possibility of directly treating the inciting factor remains attractive and further studies are warranted. In cerebral stroke, neuroprotective agents have been advocated, but studies in SCInf are lacking.^65^ Similarly, immune modulation may play a role in future care.^60^

The relatively good potential for functional recovery, including ambulation, indicates the need for specialised multidisciplinary rehabilitation services with the capacity to manage patients in the long term.^38^

## Supplementary material

10.1136/bmjno-2024-000754 online supplemental file 1

## Data Availability

Data sharing not applicable as no datasets generated and/or analysed for this study.
