# Supplementary material for: Spontaneous spinal cord infarction: a systematic review
Source: BMJ Neurol Open. 2024 May 27;6(1):e000754. doi: 10.1136/bmjno-2024-000754 (PMC11138300; doi:10.1136/bmjno-2024-000754)
Supplement: online supplemental file 1 [file bmjno-2024-000754supp001.pdf]

**Supplementary file 1**

PRISMA 2020 checklist

| Section and Topic | Item # | Checklist item | Location where item is reported |
|---|---|---|---|
| TITLE | | | |
| Title | 1 | Identify the report as a systematic review. | In the title |
| ABSTRACT | | | |
| Abstract | 2 | See the PRISMA 2020 for Abstracts checklist. | In the abstract |
| INTRODUCTION | | | |
| Rationale | 3 | Describe the rationale for the review in the context of existing knowledge. | In the “rationale” section of the introduction |
| Objectives | 4 | Provide an explicit statement of the objective(s) or question(s) the review addresses. | In the last paragraph of the introduction |
| METHODS | | | |
| Eligibility criteria | 5 | Specify the inclusion and exclusion criteria for the review and how studies were grouped for the syntheses. | In the “eligibility criteria” section of methods |
| Information sources | 6 | Specify all databases, registers, websites, organisations, reference lists and other sources searched or consulted to identify studies. Specify the date when each source was last searched or consulted. | In the “databases and search strategy” section of methods |
| Search strategy | 7 | Present the full search strategies for all databases, registers and websites, including any filters and limits used. | “Supplementary file 2” and in the “databases and search strategy” section of methods |
| Selection process | 8 | Specify the methods used to decide whether a study met the inclusion criteria of the review, including how many reviewers screened each record and each report retrieved, whether they worked independently, and if applicable, details of automation tools used in the process. | In the “study selection” section of methods |
| Data collection process | 9 | Specify the methods used to collect data from reports, including how many reviewers collected data from each report, whether they worked independently, any processes for obtaining or confirming data from study investigators, and if applicable, details of automation tools used in the process. | In the “data extraction” section of methods |
| Data items | 10a | List and define all outcomes for which data were sought. Specify whether all results that were compatible with each outcome domain in each study were sought (e.g. for all measures, time points, analyses), and if not, the methods used to decide which results to collect. | In the “data extraction” section of methods |

| Section and Topic | Item # | Checklist item | Location where item is reported |
|---|---|---|---|
| | 10b | List and define all other variables for which data were sought (e.g. participant and intervention characteristics, funding sources). Describe any assumptions made about any missing or unclear information. | In the “data extraction” section of methods |
| Study risk of bias assessment | 11 | Specify the methods used to assess risk of bias in the included studies, including details of the tool(s) used, how many reviewers assessed each study and whether they worked independently, and if applicable, details of automation tools used in the process. | In the “data synthesis and risk of bias assessment” section of methods |
| Effect measures | 12 | Specify for each outcome the effect measure(s) (e.g. risk ratio, mean difference) used in the synthesis or presentation of results. | In the “data synthesis and risk of bias assessment” section of methods |
| Synthesis methods | 13a | Describe the processes used to decide which studies were eligible for each synthesis (e.g. tabulating the study intervention characteristics and comparing against the planned groups for each synthesis (item #5)). | In the “eligibility criteria” section of methods |
| | 13b | Describe any methods required to prepare the data for presentation or synthesis, such as handling of missing summary statistics, or data conversions. | n/a |
| | 13c | Describe any methods used to tabulate or visually display results of individual studies and syntheses. | n/a |
| | 13d | Describe any methods used to synthesize results and provide a rationale for the choice(s). If meta-analysis was performed, describe the model(s), method(s) to identify the presence and extent of statistical heterogeneity, and software package(s) used. | n/a |
| | 13e | Describe any methods used to explore possible causes of heterogeneity among study results (e.g. subgroup analysis, meta-regression). | n/a |
| | 13f | Describe any sensitivity analyses conducted to assess robustness of the synthesized results. | n/a |
| Reporting bias assessment | 14 | Describe any methods used to assess risk of bias due to missing results in a synthesis (arising from reporting biases). | In the “data synthesis and risk of bias assessment” section of methods |
| Certainty assessment | 15 | Describe any methods used to assess certainty (or confidence) in the body of evidence for an outcome. | n/a |
| RESULTS | | | |
| Study | 16a | Describe the results of the search and selection process, from the number of records identified in the search to the number of studies included in the review, ideally using a flow | In the “characteristics of the included studies” |

| Section and Topic | Item # | Checklist item | Location where item is reported |
|---|---|---|---|
| selection | | diagram. | section of the results |
| | 16b | Cite studies that might appear to meet the inclusion criteria, but which were excluded, and explain why they were excluded. | n/a |
| Study characteristics | 17 | Cite each included study and present its characteristics. | In the “characteristics of the included studies” section of the results |
| Risk of bias in studies | 18 | Present assessments of risk of bias for each included study. | In the “characteristics of the included studies” section of the results |
| Results of individual studies | 19 | For all outcomes, present, for each study: (a) summary statistics for each group (where appropriate) and (b) an effect estimate and its precision (e.g. confidence/credible interval), ideally using structured tables or plots. | n/a |
| Results of syntheses | 20a | For each synthesis, briefly summarise the characteristics and risk of bias among contributing studies. | n/a |
| | 20b | Present results of all statistical syntheses conducted. If meta-analysis was done, present for each the summary estimate and its precision (e.g. confidence/credible interval) and measures of statistical heterogeneity. If comparing groups, describe the direction of the effect. | n/a |
| | 20c | Present results of all investigations of possible causes of heterogeneity among study results. | n/a |
| | 20d | Present results of all sensitivity analyses conducted to assess the robustness of the synthesized results. | n/a |
| Reporting biases | 21 | Present assessments of risk of bias due to missing results (arising from reporting biases) for each synthesis assessed. | In the “characteristics of the included studies” section of the results |
| Certainty of evidence | 22 | Present assessments of certainty (or confidence) in the body of evidence for each outcome assessed. | n/a |
| DISCUSSION | | | |
| Discussion | 23a | Provide a general interpretation of the results in the context of other evidence. | In the first paragraph of the discussion |
| | 23b | Discuss any limitations of the evidence included in the review. | In the “limitation” section of the discussion |
| | 23c | Discuss any limitations of the review processes used. | In the “limitation” section of the discussion |

| Section and Topic | Item # | Checklist item | Location where item is reported |
|---|---|---|---|
| | 23d | Discuss implications of the results for practice, policy, and future research. | In the “future perspectives” section of the discussion |
| OTHER INFORMATION | | | |
| Registration and protocol | 24a | Provide registration information for the review, including register name and registration number, or state that the review was not registered. | In the first paragraph of the methods |
| | 24b | Indicate where the review protocol can be accessed, or state that a protocol was not prepared. | In the first paragraph of the methods |
| | 24c | Describe and explain any amendments to information provided at registration or in the protocol. | In the first paragraph of the methods |
| Support | 25 | Describe sources of financial or non-financial support for the review, and the role of the funders or sponsors in the review. | In the “funding” section at the end of the review |
| Competing interests | 26 | Declare any competing interests of review authors. | In the “conflict of interest” section at the end of the review |
| Availability of data, code and other materials | 27 | Report which of the following are publicly available and where they can be found: template data collection forms; data extracted from included studies; data used for all analyses; analytic code; any other materials used in the review. | In the “data availability statement” section at the end of the review |

*From:* Page MJ, McKenzie JE, Bossuyt PM, Boutron I, Hoffmann TC, Mulrow CD, et al. The PRISMA 2020 statement: an updated guideline for reporting systematic reviews. BMJ 2021;372:n71. doi: 10.1136/bmj.n71

For more information, visit: http://www.prisma-statement.org/

Supplementary file 2: planned search strategy

| **Database** | **Search strategy** |
|---|---|
| PubMed | ((idiopathic OR spontaneous OR "non-surgical" OR "nonsurgical" OR "non-iatrogenic" OR "non iatrogenic" OR "noniatrogenic" OR "non-traumatic" OR "nontraumatic") AND ("spinal cord isch*" OR "spinal cord infarct*" OR "spinal isch*" OR "spinal infarct*")) OR ("spinal cord stroke" OR "spinal stroke" OR ”spinal artery infarct*” OR ”spinal artery isch*”) Filters: English |
| Web of Science | ((idiopathic OR spontaneous OR "non-surgical" OR "nonsurgical" OR "non-iatrogenic" OR "non iatrogenic" OR "noniatrogenic" OR "non-traumatic" OR "nontraumatic") AND ("spinal cord isch*" OR "spinal cord infarct*" OR "spinal isch*" OR "spinal infarct*")) OR ("spinal cord stroke" OR "spinal stroke" OR ”spinal artery infarct*” OR ”spinal artery isch*”) (All Fields) and English (Languages) |
| Embase | ((idiopathic OR spontaneous OR 'non-surgical' OR 'nonsurgical' OR 'non-iatrogenic' OR 'non iatrogenic' OR 'noniatrogenic' OR 'non-traumatic' OR 'nontraumatic') AND ('spinal cord isch*' OR 'spinal cord infarct*' OR 'spinal isch*' OR 'spinal infarct*') OR 'spinal cord stroke' OR 'spinal stroke' OR 'spinal artery infarction' OR 'spinal artery ischemia' OR 'spinal artery ischaemia') AND ([article]/lim OR [article in press]/lim OR [data papers]/lim OR [erratum]/lim OR [review]/lim) AND [humans]/lim AND [english]/lim AND [clinical study]/lim |

## Supplementary file 3

Risk of bias assessment according to the Newcastle-Ottawa Scale

| Study ID | Representativeness of intervention group (1p) | Selection of the non exposed cohort (1p) | Ascertainment of exposure (1p) | Demonstration that outcome of interest was not present at start of study (1p) | Comparability of cohorts on the basis of the design or analysis (2p) | Assessment of outcome (1p) | Was follow-up long enough for outcomes to occur (1p) | Adequacy of follow up of cohorts (1p) | Overall score (9p) |
|---|---|---|---|---|---|---|---|---|---|
| Elksnis 1991 | 0 | 0 | 1 | 1 | 0 | 1 | 0 | 1 | 4 |
| Monteiro 1992 | 0 | 0 | 1 | 1 | 0 | 1 | 0 | 1 | 4 |
| Yuh 1992 | 0 | 0 | 1 | 1 | 0 | 1 | 0 | 1 | 4 |
| Pelser 1993 | 0 | 0 | 1 | 1 | 0 | 1 | 1 | 1 | 5 |
| Cheshire 1996 | 1 | 0 | 1 | 1 | 0 | 1 | 0 | 1 | 5 |
| Faig 1998 | 0 | 0 | 1 | 1 | 0 | 1 | 0 | 1 | 4 |
| Kim 1988 | 1 | 0 | 1 | 1 | 0 | 1 | 0 | 0 | 4 |
| Weidauer 2002 | 0 | 0 | 1 | 1 | 0 | 1 | 0 | 0 | 3 |
| Thurnher 2006 | 0 | 0 | 1 | 1 | 0 | 1 | 0 | 0 | 3 |
| Srikanth 2007 | 0 | 0 | 1 | 1 | 0 | 1 | 0 | 0 | 3 |
| Cheng 2008 | 1 | 0 | 1 | 1 | 0 | 1 | 1 | 1 | 6 |
| Ghandehari 2010 | 1 | 0 | 1 | 1 | 0 | 1 | 0 | 0 | 4 |
| Struhal 2011 | 0 | 0 | 1 | 1 | 0 | 1 | 0 | 1 | 4 |
| Alblas 2012 | 0 | 0 | 1 | 1 | 0 | 1 | 0 | 1 | 4 |
| New 2012 | 1 | 0 | 1 | 1 | 0 | 1 | 0 | 0 | 4 |
| Robertson 2012 | 1 | 0 | 1 | 1 | 0 | 1 | 1 | 1 | 6 |
| New 2013 | 1 | 0 | 1 | 1 | 0 | 1 | 1 | 1 | 6 |
| Spencer 2014 | 0 | 0 | 1 | 1 | 0 | 1 | 1 | 1 | 5 |
| Rigney 2015 | 0 | 0 | 1 | 1 | 0 | 1 | 0 | 1 | 4 |
| Artemis 2017 | 0 | 0 | 1 | 1 | 0 | 1 | 0 | 0 | 3 |
| Bar 2017 | 0 | 0 | 1 | 1 | 0 | 1 | 1 | 1 | 5 |
| Pikija 2017 | 1 | 0 | 1 | 1 | 0 | 1 | 0 | 0 | 4 |
| Qureshi 2017 | 0 | 0 | 1 | 1 | 0 | 1 | 0 | 1 | 4 |
| Yadav 2018 | 0 | 0 | 1 | 1 | 0 | 1 | 0 | 0 | 3 |
| Zalewski 2018 | 1 | 0 | 1 | 1 | 0 | 1 | 0 | 1 | 5 |
| Zalewski 2019 | 1 | 0 | 1 | 1 | 0 | 1 | 0 | 1 | 5 |

| | | | | | | | | | |
|---|---|---|---|---|---|---|---|---|---|
| Ashton 2020 | 0 | 0 | 1 | 1 | 0 | 1 | 0 | 1 | 4 |
| English 2020 | 1 | 0 | 1 | 1 | 0 | 1 | 0 | 0 | 4 |
| Ge 2020 | 0 | 0 | 1 | 1 | 0 | 1 | 0 | 0 | 3 |
| Park 2020 | 0 | 0 | 1 | 1 | 0 | 1 | 0 | 1 | 4 |
| Sechi 2021 | 1 | 0 | 1 | 1 | 0 | 1 | 0 | 0 | 4 |
| Pikija 2022 | 1 | 0 | 1 | 1 | 0 | 1 | 0 | 0 | 4 |
| Stenimahitis 2023 | 1 | 0 | 1 | 1 | 0 | 1 | 1 | 1 | 6 |
